# Hydrogen Sulfide and Endothelium-Dependent Vasorelaxation

**DOI:** 10.3390/molecules191221183

**Published:** 2014-12-16

**Authors:** Jerzy Bełtowski, Anna Jamroz-Wiśniewska

**Affiliations:** 1 Department of Pathophysiology, Medical University, 20-150 Lublin, Poland; 2 Department of Neurology, Medical University, 20-090 Lublin, Poland; E-Mail: ajamroz@wp.pl

**Keywords:** hydrogen sulfide, endothelium-derived hyperpolarizing factor, potassium channels, nitric oxide, cyclic GMP, protein kinase G, nitroxyl, perivascular sensory neurons

## Abstract

In addition to nitric oxide and carbon monoxide, hydrogen sulfide (H_2_S), synthesized enzymatically from l-cysteine or l-homocysteine, is the third gasotransmitter in mammals. Endogenous H_2_S is involved in the regulation of many physiological processes, including vascular tone. Although initially it was suggested that in the vascular wall H_2_S is synthesized only by smooth muscle cells and relaxes them by activating ATP-sensitive potassium channels, more recent studies indicate that H_2_S is synthesized in endothelial cells as well. Endothelial H_2_S production is stimulated by many factors, including acetylcholine, shear stress, adipose tissue hormone leptin, estrogens and plant flavonoids. In some vascular preparations H_2_S plays a role of endothelium-derived hyperpolarizing factor by activating small and intermediate-conductance calcium-activated potassium channels. Endothelial H_2_S signaling is up-regulated in some pathologies, such as obesity and cerebral ischemia-reperfusion. In addition, H_2_S activates endothelial NO synthase and inhibits cGMP degradation by phosphodiesterase 5 thus potentiating the effect of NO-cGMP pathway. Moreover, H_2_S-derived polysulfides directly activate protein kinase G. Finally, H_2_S interacts with NO to form nitroxyl (HNO)—a potent vasorelaxant. H_2_S appears to play an important and multidimensional role in endothelium-dependent vasorelaxation.

## 1. Introduction

It was first proposed by Abe and Kimura almost two decades ago that hydrogen sulfide (H_2_S) is the endogenous mediator in mammals [1]. Since that time this hypothesis was confirmed by many studies and the “H_2_S field” in biology and medicine is now growing rapidly. H_2_S thus joined two older counterparts, nitric oxide (NO) and carbon monoxide (CO), to form the family of “gasotransmitters” [2]. Other gasotransmitters, such as ammonia (NH_3_), methane (CH_4_) and hydrogen (H_2_), are suggested to exist as well [3]. H_2_S is now well-recognized to be involved in the regulation of cardiovascular system, inflammatory and immune response, gastrointestinal tract, kidney and nervous system functions [4,5]. Alterations of the “H_2_S pathway” have been observed in many diseases and H_2_S donors or, on the contrary, inhibitors of H_2_S-synthesizing enzymes, are now considered as the potential therapeutic agents for the treatment of these diseases [6,7].

Effect of H_2_S on vascular tone was one of the first biological activities of this gasotransmitter described in the literature [8]. Initial studies suggested that in the vascular wall H_2_S is produced only by smooth muscle cells (SMCs) and induces vasorelaxation by activating ATP-sensitive potassium channels in these cells [9]. However, now it is clear that H_2_S may be produced in other layers of the vascular wall including endothelial cells and perivascular adipose tissue (PVAT) as well. Role of SMC- and PVAT-derived H_2_S in the regulation of vascular tone in physiological and pathological conditions was described in details in many review articles [10,11,12,13,14]. Herein we focus on the role of H_2_S in endothelium-dependent vasorelaxation, which is a relatively new topic.

## 2. Endothelium as the Regulator of Vascular Tone

Endothelium is a single layer of cells, which cover the internal surface of all blood vessels in the human body. Its thickness is from 1 μm in large arteries and veins to 0.1 μm in the microvasculature, its total surface ranges from 300 to 1000 m^2^ and total weight is about 1.5 kg. Healthy endothelium plays many important roles in the vascular system including vasorelaxation, inhibition of platelet aggregation and blood clotting, inhibiting adhesion and migration of leukocytes and suppressing smooth muscle cells proliferation. Endothelial dysfunction may be induced by many factors, such as oxidative stress, hyperglycemia, hyperlipidemia, smoking or elevated homocysteine concentration, and contributes to the development of various cardiovascular diseases, in particular arterial hypertension and atherosclerosis [15].

In response to many stimuli, such as shear stress, circulating hormones (insulin, estradiol), neurotransmitters (acetylcholine), autacoids (bradykinin), and growth factors, such as vascular endothelial growth factor (VEGF), endothelial cells secrete mediators, which regulate vascular tone. The first of them which was identified, nitric oxide (NO), is synthesized from l-arginine by endothelial NO synthase (eNOS, NOS3) and relaxes vascular smooth muscle cells by stimulating soluble guanylyl cyclase (sGC) and increasing intracellular cyclic guanosine 3',5'-monophoshpate (cGMP), which activates protein kinase G (PKG) [16]. eNOS activity is stimulated by the increase in intracellular Ca^2+^ concentration in a calmodulin-dependent manner in response to various factors, such as acetylcholine, bradykinin or substance P. Ca^2+^/calmodulin-dependent stimulation, is a rapid but relatively short-lasting mechanisms. In addition, eNOS activity may be regulated in a more prolonged manner by phosphorylation; most of these phosphorylations, such as at Ser^1177^, are stimulatory but some of them decrease enzyme activity. Phosphorylation of eNOS is catalyzed by various protein kinases, such as protein kinase B/Akt, AMP-stimulated protein kinase (AMPK) and p38 mitogen-activated protein kinase (p38 MAPK) [17]. The eNOS-NO-cGMP-PKG pathway is the best characterized mechanism of endothelium-dependent vasorelaxation; its importance in the regulation of vascular tone is evidenced by rapid increase in blood pressure after administration of NO synthase inhibitors as well as severe arterial hypertension in eNOS knockout mice. This pathway is also targeted by some currently used drugs, such as organic nitrates (NO donors), inhibitors of cGMP-metabolizing phosphodiesterase, such as sildenafil and, most recently, direct NO-independent sGC activators. However, the NO-cGMP pathway may be compromised by many factors, such as reduced expression/activity of eNOS, deficiency of its substrate, l-arginine, or its cofactor, tetrahydrobiopterin (BH_4_), enzyme uncoupling leading to generation of superoxide anion radical instead of NO, elevated concentration of endogenous eNOS inhibitor, asymmetric dimethylarginine (ADMA) and, finally, scavenging of NO by reactive oxygen species. Consequently, endothelial dysfunction, defined as the impairment of vasorelaxation in response to eNOS-activating mediators, such as acetylcholine is observed in multiple human diseases as well as in their experimental animal models [18]. Because NO not only induces vasorelaxation but also inhibits vascular inflammatory reaction, platelet aggregation and smooth muscle cell migration and proliferation, its deficiency contributes not only to the development of diseases associated with increased vascular resistance, such as systemic or pulmonary arterial hypertension, but also to atherosclerosis and restenosis after vascular angioplasty.

The second mechanism of endothelium-dependent vasorelaxation is initiated by synthesis of prostacyclin (PGI_2_) by cyclooxygenase (COX); PGI_2_ then relaxes smooth muscle cells by activating plasma membrane receptors coupled to adenylate cyclase and increasing intracellular cAMP concentration. Since the beginning of studies focused on endothelium-dependent vasorelaxation it became evident that a portion of it resistant to both eNOS and COX inhibition exists in many vascular preparations. This mechanism is referred to as endothelium-dependent hyperpolarizing factor (EDHF) because is associated with hyperpolarization of smooth muscle cells and, as evidenced more recently, endothelial cells. Over time, multiple substances have been suggested as the candidates for EDHF including cytochrome P450-dependent arachidonate derivatives, epoxyeicosatrienoic acids (EETs), hydrogen peroxide (H_2_O_2_), C-type natriuretic peptide (CNP), carbon monoxide (CO) or even NO itself [19,20,21]. Now it is evident that there is no one universal EDHF but every of listed factors may play this role depending on species, vascular bed and experimental conditions. In addition, stimulation of endothelial small and intermediate-conductance Ca^2+^-activated potassium channels, SK_Ca_ (K_Ca_ 2.3) and IK_Ca_ (K_Ca_ 3.1), respectively, induces EC hyperpolarization, which is directly transferred to smooth muscle cells through the myoendothelial gap junctions (MEGJ). Potassium efflux through SK_Ca_ and IK_Ca_ increases extracellular K^+^ concentration at the interface between ECs and SMCs, which stimulates inwardly-rectifying potassium channels (K_ir_) or Na^+^,K^+^-ATPase. Potassium influx to smooth muscle cells by one of both of these transporters results in SMCs hyperpolarization, decrease in Ca^+^ influx through voltage-sensitive calcium channels, and vasorelaxation [22,23].

## 3. H_2_S—Synthesis and Function in the Blood Vessels

H_2_S is synthesized by three enzymes: cystathionine β-synthase (CBS), cystathionine γ-lyase (CSE) and 3-mercaptopyruvate sulfurtransferase (3-MST) in cooperation with cysteine aminotransferase (aspartate aminotransferase) [24]. CBS and CSE are enzymes of the transsulfuration pathway in which homocysteine is metabolized to cysteine. CBS catalyzes the reaction between homocysteine and serine to form cystathionine and H_2_O, whereas CSE breaks down cystathionine to cysteine, ammonia and 2-ketobutyrate. H_2_S may be synthesized by these enzymes in alternative reactions [25,26]. In particular, in the reaction catalyzed by CBS serine may be replaced by cysteine with cystathionine and H_2_S being the products. CSE may catalyze β-elimination of cysteine to pyruvate, H_2_S and NH_4_^+^, γ-elimination of homocysteine to 2-ketobutyrate, H_2_S and NH_4_^+^ and β- or γ-replacement reaction between two cysteine or two homocysteine molecules, with lanthionine or homolanthionine, respectively, as the co-products [25,26].

The steady-state H_2_S concentration in tissues is very low because it is rapidly metabolized in mitochondria. H_2_S is first oxidized to persulfide sulfur by sulfide:quinone oxidoreductase (SQR) which transfers electrons from H_2_S to ubiquinone where they enter mitochondrial respiratory chain. Further steps of H_2_S metabolism are catalyzed by persulfide dioxygenase (ETHE1; enzyme deficient in rare inherited disorder, methylmalonic encephalopathy), thiosulfate:cyanide sulfurtransferase (rhodanese) and sulfite oxidase, with thiosulfate (SSO_3_^2−^) and sulfate (SO_4_^2−^) being the final products [27]. H_2_S oxidation is highly dependent on oxygen level. It is suggested that H_2_S signaling is low under normoxic conditions but increases markedly in hypoxic tissues due to reduced H_2_S oxidation; sulfide is thus considered to be the ”oxygen sensor” which mediates tissues response to hypoxia [28].

Several signaling mechanisms of H_2_S have been described, such as reaction with heme-containing proteins, protein *S*-sulfhydration (conversion of cysteine thiol -SH to persulfide -SSH groups), reaction with reactive oxygen species, or NO and related species as well as reduction of protein disulfide bonds to thiols [29]. Recent studies indicate that protein *S*-sulfhydration is not mediated by H_2_S itself but rather by its more oxidized species, polysulfides (H_2_S_n_). These issues are outside the scope of this review; they are covered by other papers in this special issue [4,30].

## 4. H_2_S as the Endothelium-Derived Hyperpolarizing Factor

NO and PGI_2_ operate as endothelium-derived relaxing factors mainly in large conduit arteries. Their contribution decreases with decreasing vessel diameter and they are progressively replaced by EDHF in small resistance vessels which are more important for the regulation of total peripheral resistance and blood pressure. Many studies have demonstrated that EDHF is inhibited by NO under baseline conditions and becomes activated when NO pathway is inhibited or deficient. In addition, it was observed that EDHF-dependent mechanism is less sensitive than NO to factors, which induce endothelial dysfunction and is relatively well preserved in disease states [19,20,21].

Several recent studies suggest that H_2_S may play a role of EDHF in some vascular beds. First, it was demonstrated that methacholine (cholinergic agonist)-induced relaxation of mesenteric arteries is impaired in CSE^−/−^ mice [31]. CSE is expressed in mouse endothelial cells and was demonstrated to be activated by Ca^2+^/calmodulin in response to calcium ionophore or cholinergic stimulation [31]. Although this mechanism of CSE regulation has been questioned [32], these data suggest that H_2_S may be synthesized in endothelial cells. In addition, both CAT and 3-MST are expressed in rat aortic endothelial cells, and these cells can produce H_2_S from l-cysteine in the presence of 2-oxoglutarate, the co-substrate of CAT [33]. These data suggest that H_2_S may be synthesized in endothelial cells and induce relaxation of adjacent smooth muscle cells.

This issue was further addressed by Mustafa *et al*. [34]. In mice mesenteric artery rings 70%–80% of acetylcholine-induced endothelium-dependent vasorelaxation was insensitive to NOS and COX inhibition, and this EDHF-mediated component was substantially inhibited in CSE knockout mice. In addition, acetylcholine induced hyperpolarization of mesenteric artery rings (measured by intracellular potential-sensitive fluorescent probe) in wild-type, but not in CSE^−/−^ mice. Acetylcholine-induced vasorelaxation of mouse or rat mesenteric artery was partially attenuated by CSE inhibitor, propargylglycine, as well as by K_ATP_ channel antagonist, glibenclamide. NaHS induced concentration-dependent relaxation of vascular rings with intact endothelium and this effect was not altered by sGC inhibitor, ODQ or PKG inhibitor, KT5823, but was partially attenuated by the mixture of apamin and charybdotoxin, which inhibit endothelial SK_Ca_ and IK_Ca_ channels, respectively, and was partially attenuated by glibenclamide. The combination of apamin, charybdotoxin and glibenclamide completely abolished NaHS-induced vasorelaxation of mesenteric artery rings with intact endothelium. NaHS also relaxed vascular rings with denuded endothelium and this effect was completely abolished by glibenclamide but not affected by apamin and charybdotoxin [34]. Similar results were obtained by us in rat mesenteric artery when slow-releasing H_2_S donor, GYY4137, and the other IK_Ca_ inhibitor, TRAM-34, were used [35]. These data suggest that H_2_S produced in endothelial cells induces vasorelaxation by activating endothelial SK_Ca_ and IK_Ca_ channels as well as by stimulating K_ATP_ channels in smooth muscle cells. This conclusion was supported by measurement of transmembrane potential in primary culture of mice endothelial cells. Acetylcholine hyperpolarized ECs isolated from wild-type but not from CSE^−/−^ mice, which was completely blocked by apamin and charybdotoxin but not inhibited by either glibenclamide or iberiotoxin—inhibitors of ATP-sensitive and large-conductance Ca^2+^-activated K^+^ channels (BK_Ca_), respectively. In contrast, NaHS hyperpolarized ECs isolated from either wild-type or CSE^−/−^ mice to the similar extent in apamin and charybdotoxin-sensitive manner. Furthermore, NaHS increased sulfhydration of IK_Ca_ in human aortic endothelial cells [34].

These findings were further confirmed by the measurement of intracellular potential with the patch-clamp technique, which is a gold standard in electrophysiological research [36]. In the third order mouse mesenteric artery with intact endothelium, NaHS and methacholine induced hyperpolarization of smooth muscle cells, whereas propargylglycine induced depolarization; effect of methacholine was not observed in endothelium-denuded rings or in endothelium-intact rings isolated from CSE knockout mice. Similarly, hyperpolarizing effect of l-cysteine and depolarizing effect of CSE inhibitor were absent in CSE knockout mice. NaHS induced similar hyperpolarization in endothelium-intact rings of wild-type and CSE knockout mice. Apamin and charybdotoxin reduced hyperpolarizing effect of methacholine or NaHS. Membrane potential of endothelial cells was also examined in that study [36]. Either methacholine or NaHS hyperpolarized ECs in apamin+charybdotoxin-sensitive manner. Hyperpolarizing effect of methacholine and l-cysteine were absent in CSE knockout mice but hyperpolarizing effect of NaHS was preserved. Hyperpolarizing effect on endothelial cells was preserved by pre-incubation with thiol-reactive compound, methylmetanethiosulfonate, and was reversed by dithiotreitol, which suggest that it resulted from sulfhydration of K^+^ channels. Interestingly, prolonged 12 h incubation of primary EC culture with NaHS resulted in the increase in the expression of SK_Ca_. Baseline SK_Ca_ expression was reduced in endothelial cells incubated with propargylglycine as well as in endothelial cells isolated from CSE knockout mice, whereas IK_Ca_ expression was not altered by NaHS, propargylglycine or CSE knockout, suggesting that SK_Ca_ may be the major sulfide target in endothelial cells. Taken, together, these studies [34,35,36] provide strong evidence that H_2_S synthesized by CSE in endothelial cells hyperpolarizes these cells by activating SK_Ca_ and/or IK_Ca_ channels and thus induces SMCs hyperpolarization. However, it is unclear if SMC hyperpolarization results from potential transfer through myoendothelial gap junctions or potassium efflux from ECs with subsequent stimulation of SMC inwardly-rectifying K^+^ channels and/or Na^+^,K^+^-ATPase.

Ductus arteriosus connects pulmonary artery with aorta during the fetal life and enables blood shunt to bypass pulmonary circulation until after birth. Ductus arteriosus is kept patent by vasodilators, such as NO, PGI_2_ and CO; postnatal decrease in their production results in progressive vessel closure. Persistent ductus arteriosus is one of the most common congenital heart defects. EDHF is normally not detectable in ductus arteriosus until vasodilators mentioned above are not inhibited or deficient. Baragatti *et al*. [37] have demonstrated that CSE, CBS and 3-MST are expressed in the intima and media layers of the ductus arteriosus and that the expression of CSE is by 45% higher in the intima than in the media whereas the expression of CBS is by 15% higher in media than in the intima. The expression of 3-MST transcript was very low in that study [35] suggesting minor role of this enzyme in H_2_S production. Both CSE inhibitor, propargylglycine, and CBS inhibitor, aminooxyacetate, induce vasoconstriction of the ductus arteriosus and the effect of the former but not of the latter is partially attenuated in vascular rings with removed endothelium. In addition, bradykinin-induced vasorelaxation measured in the presence of NOS, COX and heme oxygenase inhibitors was inhibited by propargylglycine. These data indicate that H_2_S of endothelial origin may play an important role in maintaining patency of ductus arteriosus. Importantly, hypoxic conditions during fetal life may increase local H_2_S concentration whereas postnatal increase in tissue oxygenation could reduce H_2_S thus leading to ductus closure [37].

Han *et al*. [38] examined acetylcholine-induced relaxation of rat cerebral basilar artery and middle cerebral artery. Acetylcholine-induced relaxation of endothelium-intact rings in both preparations was partially attenuated by l-NAME and most of l-NAME-resistant component was abolished by propargylglycine, as was acetylcholine-induced hyperpolarization of smooth muscle cells. Interestingly, cerebral ischemia-reperfusion reduced the l-NAME-sensitive component of vasorelaxation but augmented propargylglycine-sensitive component and vessel hyperpolarization [38]. These data support the concept that H_2_S-dependent EDHF is a back-up mechanism of the regulation of vascular tone important when NO becomes deficient.

## 5. H_2_S and Vascular Effect of Leptin

Obesity and the accompanying metabolic syndrome are the major causes of cardiovascular diseases worldwide. Studies of the last 10–15 years suggest that an important role in obesity-associated cardiovascular diseases is played by abnormal secretion of adipose tissue hormones (adipokines) [39,40,41]. Leptin is the first and the best characterized adipokine. It is produced by white adipose tissue in amounts proportional to adiposity status and regulates energy balance by inhibiting food intake and increasing energy expenditure. Many studies have demonstrated that leptin induces endothelium-dependent and independent vasorelaxation and that this effect is impaired in obesity [42,43]. Chronic hyperleptinemia either induced in lean rats by administration of exogenous hormone or “endogenous” hyperleptinemia associated with obesity induced by highly palatable diet impairs acute vascular NO-mimetic effect of leptin [44,45]. However, in the early phase of obesity when insulin sensitivity is not impaired, total vasodilating effect of leptin is intact because NO deficiency is compensated by EDHF. During progression of the metabolic syndrome when insulin resistance develops both NO- and EDHF components are compromised [45].

Recently, we characterized vascular effect of leptin in rat mesenteric artery rings [35]. Leptin-induced endothelium-dependent vasorelaxation was partially attenuated by l-NAME but mostly abolished by apamin and TRAM-34. The apamin + TRAM-34 sensitive component was not inhibited by cytochrome P450 inhibitor (SKF 525A), lipoxygenase inhibitor nondihydroguaiateric acid, H_2_O_2_ scavenger, PEG-catalase, heme oxygenase inhibitor, (Cr(III) mesoporphyrin IX), and protein kinase G inhibitor (KT5823) which blocks natriuretic peptide signaling, indicating that neither of these putative EDHFs was involved. In contrast, H_2_S scavenger, bismuth (III) subsalicylate (BSS) or propargylglycine almost completely abolished the EDHF-dependent portion of leptin-induced vasorelaxation. In contrast, neither BSS nor propargylglycine had any effect on NO-dependent component. These data indicate that the effect of leptin is H_2_S-dependent.

Next, we aimed to examine if and how this effect of leptin is modulated in the metabolic syndrome. First, we examined acute vascular effect of leptin in rats with chronic hyperleptinemia induced by seven-day exogenous leptin administration. Total leptin-induced endothelium-dependent vasorelaxation was intact in this group, however, the l-NAME sensitive component was reduced and the apamin+TRAM-34 sensitive component was augmented. The similar effect was observed in rats made obese by feeding highly-palatable diet for four weeks. Importantly, this model is characterized by body weight gain and chronic hyperleptinemia but plasma glucose, lipoproteins, insulin concentration and insulin sensitivity are still normal. In both hyperleptinemic and obese groups, the entire EDHF component of leptin-induced vasorelaxation was mediated by H_2_S because it was abolished by either propargylglycine or BSS like in lean control group. To examine if alterations of NO and EDHF mechanisms in the obese group are accounted for by hyperleptinemia, we included additional groups treated with recently developed PEG-ylated superactive leptin receptor antagonist (PEG-SRLA). PEG-SRLA blocks the effects of endogenous leptin but does not reduce or even aggravates other disturbances observed in obesity by reducing anorectic effect of leptin and further increasing weight gain. We observed that PEG-SRLA administered for seven days to healthy control rats had no effect on leptin-induced NO and EDHF-mediated endothelium-dependent vasorelaxation but abolished decrease in NO and increase in EDHF in obese animals [35]. These results indicate that increase in EDHF/H_2_S in obese rats was accounted for by hyperleptinemia rather than other hormonal or metabolic disturbances associated with obesity.

Then we asked why H_2_S mediated effect of leptin is enhanced in hyperleptinemic and obese animals. First, we hypothesized that CSE expression or activity could be altered. However, CSE activity [35] and expression (data not published) were not different between groups. Second, we took into account the possibility that leptin-induced H_2_S signaling could be augmented due to the impairment of its mitochondrial oxidation. However, although SQR inhibitor, stigmatellin, increased the EDHF-dependent effect of leptin, the difference between lean, hyperleptinemic and obese groups was still observed. Finally, we examined the sensitivity of our vascular preparation to H_2_S. Vascular effect of GYY4137 on endothelium-denuded mesenteric artery rings was similar in lean, hyperleptinemic and obese groups, and in all these groups was completely abolished by glibenclamide suggesting that sensitivity of smooth muscle cells to H_2_S is not altered in obesity. Similarly, the effect of GYY4137 on rings with intact endothelium measured in the presence of apamin and TRAM-34 was not different. However, vasorelaxant effect of GYY4137 in rings with preserved endothelium without any inhibitors as well as its apamin + TRAM-34 sensitive component were higher in hyperleptinemic and obese than in lean animals. Recently, we have demonstrated that leptin stimulated similar increase in H_2_S production in primary culture of endothelial cells isolated from lean, hyperleptinemic and obese rats, however, leptin-induced hyperpolarization was higher in cells isolated from hyperleptinemic or obese animals (unpublished observation). These data suggest that either the expression of SK_Ca_ and/or IK_Ca_ channels is increased or that these channels are more sensitive to H_2_S. Previously, it has been demonstrated that IK_Ca_ activator, 1-EBIO, produces more marked vasorelaxation of the fourth order mesenteric artery isolated from rats made obese by high-fat diet than in arteries of lean animals, and that IK_Ca_ expression was higher in obese rats [46]. In addition, because protein sulfhydration is induced not by H_2_S itself but rather by more oxidized sulfide species, such as polysulfides, oxidative stress induced by hyperleptinemia and/or obesity, could render these channels more sensitive to H_2_S-induced modification.

## 6. Is eNOS-NO-cGMP System Involved in Vascular Effect of H_2_S?

In the first study in which vascular effect of H_2_S was characterized in more detail, Zhao *et al*. [9] reported that l-NAME reduced sensitivity of rat aortic rings to vasorelaxant effect of NaHS suggesting some cooperation between H_2_S and NO. However, sGC inhibitor, ODQ, had no effect on NaHS-induced relaxation. Interestingly, in the subsequent study, the same group observed that two distinct sGC inhibitors, ODQ and NS-2028, tended to potentiate NaHS-induced vasorelaxation [47].

Several studies have demonstrated that NaHS inhibits arginine uptake and eNOS activity in endothelial cells [48,49,50]. However, in these studies relatively high NaHS concentrations and/or incubation times were used. Because NaHS rapidly oxidizes in solution to other sulfur species, the mechanism of these effects and their physiological relevance is not clear. In contrast, more recent studies indicate that H_2_S may positively cooperate with NO in the vascular system. In cultured bovine aortic endothelial cells (BAECs), Na_2_S (100 μM) induced a time-dependent increase in NO production measured by the spin-trap method with maximal almost 90% stimulation observed after 30 min [51]. Na_2_S had no effect on total eNOS expression but increased the amount of enzyme phosphorylated at Ser^1177^ by 140%. Effect on eNOS phosphorylation and NO production were abolished by Akt inhibitor, triciribine.

Concentration-dependent stimulatory effect of NaHS (25–300 μM) on NO production (measured as accumulation of nitrites and nitrates in the culture medium) in BAECs was also observed by Kida *et al.* [52]. This effect was abolished by intracellular calcium chelator, BAPTA-AM, but not by removing Ca^2+^ from the extracellular fluid, and was attenuated by inhibitor of endoplasmic reticulum ryanodine receptor (RyR), dantrolene, and inositol triphosphate receptor antagonist, xestospongin. In addition, treatment with NaHS increased intracellular Ca^2+^ concentration in dantrolene- and xestospsongin-sensitive manner. These data indicate that calcium release from endoplasmic reticulum is involved in H_2_S-induced eNOS activation. Moreover, NaHS increased Akt phosphorylation at Ser^473^ followed by the increase in eNOS phosphorylation at Ser^1177^ [52]. The effect on Akt phosphorylation was abolished by phosphoinositide 3-kinase (PI3K) inhibitor, wortmannin, which, however, had no effect on eNOS phosphorylation and NO production. These results indicate that although PI3K is involved in NaHS-induced increase in Akt phosphorylation, eNOS phosphorylation is PI3K-Akt independent. It is suggested that eNOS phosphorylation at Ser^1177^ might be mediated by calcium-calmodulin dependent protein kinase [52].

Subsequently, Coletta *et al*. [53] examined the role of NO in H_2_S-induced proliferation and migration (processes important for angiogenesis) of mouse brain microvascular endothelial cells bEnd3. Both H_2_S and l-cysteine stimulated angiogenesis in l-NAME and ODQ-sensitive manner. In addition, angiogenic effect of NaHS was abolished in eNOS knockout mice. On the other hand, silencing of the CSE-encoding gene abolished angiogenic effect of not only l-cysteine but also the NO donor, DEA NONOate These data suggest that NO is required for angiogenic effect of H_2_S and, *vice versa*, H_2_S is required for angiogenic effect of NO. In addition, NaHS increased cGMP concentration in endothelial cells and this effect was inhibited by l-NAME or ODQ. NaHS had no effect on purified sGC and did not enhance its stimulation by DEA NONOate. However, NaHS dose-dependently reduced PDE5A activity at relatively low concentrations (1–10 μM). The effect of 10 μM NaHS was comparable to that of synthetic PDE5 inhibitor, sildenafil. Moreover, NaHS increased Akt phosphorylation at Ser^493^ and eNOS phosphorylation at Ser^1177^, and reduced eNOS phosphorylation at Thr^495^; phosphorylation of this residue decreases enzyme activity. Either NaHS or DEA NONOate increased VASP phosphorylation at Ser^239^, which is commonly used marker of protein kinase G activity. The relevance of H_2_S-NO cooperation for the regulation of vascular tone was supported by the finding that CSE knockdown attenuated relaxing effects of acetylcholine and DEA NONOate as well as stimulatory effect of NO donor on VASP phosphorylation in rat aortic rings [53]. NaHS restored acetylcholine-induced relaxation and cGMP accumulation in vascular rings lacking CSE, and synthetic cGMP analogue, 8-bromo-cGMP, restored vasorelaxant effect of acetylcholine as well. Moreover, vasodilating effect of NaHS was impaired by l-NAME, ODQ or PKG inhibitor, and is reduced in eNOS^−/−^ mice. Low (per se non-vasorelaxant) concentration of NaHS potentiated vasodilating effects of acetylcholine and DEA NONOate, whereas CSE silencing reduced the effect of NO donor on cGMP accumulation, VASP phosphorylation and vascular tone. Taken together, these data suggest that: (1) H_2_S may stimulate the NO-cGMP pathway by increasing eNOS activity and inhibiting cGMP-degrading phosphodiesterase; (2) the NO-cGMP pathway contributes to proangiogenic and vasodilating effect of H_2_S; (3) H_2_S facilitates vascular effect of NO possibly by inhibiting cGMP degradation.

NaHS- and l-cysteine-induced increase in NO production was also observed in human umbilical vein endothelial cells [54]. In addition, CSE knockdown decreased and CSE overexpression increased intracellular NO measured with the DAF-FM fluorescent probe and extracellular accumulation of nitrites and nitrates. Consistently with previously discussed studies, NaHS increased eNOS phosphorylation at Ser^1177^, which was abolished by p38 MAPK or PI3K inhibitors, SB202198 and LY294002, respectively. Although NaHS increased phosphorylation of extracellular signal-regulated kinases (ERK) as well, these kinases were not involved in eNOS phosphorylation as evidenced by no effect of their inhibitor. SB202198 and LY294002 used together (but neither of them alone) abolished the effect of NaHS on NO production [54].

CSE knockout mice are characterized by decrease in eNOS phosphorylation at Ser^1177^, increase in inhibitory enzyme phosphorylation at Thre^493^ and decrease in myocardial BH_4_ concentration. Total and phosphorylated Akt concentrations did not differ between wild-type and CSE knockout mice, but plasma and myocardial NO metabolites and cGMP were lower in mice lacking CSE [55]. Both Na_2_S and diallyltrisulfide (DATS) reduced infarct area in the heart of wild-type mice subjected to ischemia-reperfusion, but this protective effect was lost in eNOS knockout mice as well as in eNOS knockout mice expressing nonphosphorylatable eNOS (with Ser^1177^ replaced by alanine) [55].

The H_2_S-mediated regulation of eNOS in primary culture of mouse endothelial cells was recently addressed in the elegant study by Altaany *et al.* [56]. These authors have demonstrated that eNOS is either *S*-sulfhydrated or *S*-nitrosylated at the same cysteine residue (Cys^443^). Under baseline conditions, 30% of enzyme is nitrosylated and 12% is *S*-sulfhydrated. NaHS increases *S*-sulfhydration and reduces *S*-nitrosylation both *in vitro* and *in vivo*. Whereas NaHS increases eNOS sulfhydration in both wild-type and CSE knockout mice, l-cysteine produces the same effect only in wild-type animals. CSE knockout mice exhibit no eNOS sulfhydration in the aortic wall and reduced NO concentration in this tissue. The NO donor, nitrosoglutathione (GSNO) increases eNOS nitrosylation, however, its effect is abolished by NaHS. However, GSNO does not abolish NaHS-induced *S*-sulfhydration. Whereas *S*-sulfhydration stimulates eNOS, *S*-nitrosylation decreases enzyme activity. These modifications of Cys^443^ have no effect on enzyme phosphorylation at Ser^1177^ and *vice versa*, phosphorylation of this serine residues does not affect either *S*-sulfhydration or *S*-nitrosylation of Cys^443^. Sulfhydration of Cys^443^ increases eNOS dimerization, whereas CSE knockout mice are characterized by sevenfold lower eNOS dimer/monomer ratio. In contrast, GSNO increases eNOS monomers. Dimerized enzyme is more effective in NO production whereas monomeric eNOS is “uncoupled”, that is produces superoxide rather than NO. Endothelial cells of CSE knockout mice produce less NO and more O_2_^−^, which is reversed by NaHS [56]. This study provides the novel interesting mechanism of eNOS regulation in endothelial cells and suggests the mechanism through which H_2_S deficiency increases and H_2_S donors reduce oxidative stress.

The role of NO-cGMP pathway in vascular effect of H_2_S is also supported by findings obtained in PKG knockout mice [57]. Aortic rings isolated from PKG-1^−/−^ mice exhibit impaired relaxation (rightward shift of the concentration-response curve) in response to NaHS. On the other hand, CSE^−/−^ mice are characterized by lower cGMP concentration in plasma, aorta and mesenteric artery, and sodium nitropruside fails to increase cGMP concentration in the mesenteric artery of CSE^−/−^ mice [57]. l-cysteine-induced vasorelaxation was also reduced in PKG-1 knockout mice although the expression of CSE in the vascular wall was not different between wild-type and PKG-1 knockout mice. It was confirmed in this study that NaHS increased PKG-dependent VASP phosphorylation at Ser^239^ in the vascular wall, and that peptide PKG inhibitor, DT-2, attenuated NaHS-induced vasorelaxation. *In vivo*, PKG inhibitor attenuated blood pressure-lowering effect of NaHS. Interestingly, GYY4137 increased cGMP concentration in aortic smooth muscle cells to a much lesser extent than NaHS. GYY4137-induced vasorelaxation was slower and not inhibited by DT-2. It is unclear if these differences between effects of NaHS and GYY4137 result from different time profiles of H_2_S release, easier oxidation of NaHS or H_2_S-independent effects of GYY4137 and/or its degradation product.

## 7. Direct Stimulation of PKG by H_2_S

Protein kinase G may be activated by hydrogen peroxide, which oxidizes thiol group of Cys^42^ and induces disulfide-linked enzyme homodimerization [58,59]. This mechanism may be involved in H_2_O_2_-dependent portion of EDHF response in some vascular preparations. Mice with Cys^42^ of PKG replaced by serine are hypertensive and exhibit impaired EDHF-mediated vasorelaxation in their resistance vessels. Recently, Stubbert *at al*. demonstrated that if NaHS was incubated with oxygen or H_2_O_2_, it was oxidized to polysulfides, which stimulated PKG in the similar manner [60]. In the presence of oxygen, NAHS oxidized and activated PKG in vascular smooth muscle cells and mouse mesenteric arteries. Mice expressing C42S PKG variant exhibited impairment of NaHS-induced relaxation of their mesenteric arteries. It is estimated that about 20% of NaHS-induced relaxation is accounted for by this mechanism. Potassium polysulfide relaxed mesenteric arteries of wild type but not of C42S mutant mice. In vivo, NaHS infused by the osmotic minipump induced nocturnal decrease in blood pressure in wild-type but not in PKG C42S mutant mice. Interestingly, although intracellular concentration of reduced glutathione (GSH) is very high, it did not interfere with polysulfide-induced PKG oxidation because, due to high pK_a_ value (about 9.0) glutathione exists mainly in the protonated form whereas dissociated form of the thiol group (-S^−^) is more susceptible to oxidation. Recent studies suggest that many effects of H_2_S may be in fact mediated by polysulfides and therefore the above-mentioned mechanism may be relevant for vascular effects of H_2_S. In addition, because H_2_O_2_ seems to be rate-limiting in polysulfide formation, these findings may explain vasodilating effect of hydrogen peroxide observed in many studies.

## 8. Role of Nitroxyl (HNO) in Vascular Effect of H_2_S

It is suggested that nitroxyl (HNO), the reduced form of nitric oxide, may be involved in the regulation of vascular tone [61,62]. In contrast to NO, HNO is a less potent sGC activator but more potent in nitrosothiol formation [63,64]. H_2_S can react with NO to form nitroxyl [65,66]. HNO induces release of calcitonin gene-related peptide (CGRP)—the well-known vasodilator—from perivascular neurons by activating transient receptor potential ankyrin (TRPA1) channel [67]. It has been demonstrated that HNO is formed intracellularly if cells are incubated with H_2_S solution, and this effect is not observed in cells treated with NO synthase inhibitors or NO scavengers. The HNO donor, Angeli’s salt (Na_2_N_2_O_3_) administered topically increased meningeal blood flow in the rat, and this effect was markedly attenuated by TRPA1 antagonist HC030311 as well as by CGRP receptor antagonist, CGRP_8–37_. In addition, vasodilating effect of Angeli’s salt *in vitro* and hypotensive effect *in vivo* are reduced in TRPA1 knockout mice. Interestingly, HNO is spontaneously formed in solution containing low amounts of NO and H_2_S (2 μM each) as well as in neurons treated with DEA NONOate and H_2_S solution. Inhibiting H_2_S-generating enzymes or NO synthase reduced baseline intracellular level of HNO [67]. In isolated mouse heart the mixture of NO and H_2_S but neither of these gasotransmitter alone increased CGRP release from chemosensory afferent fibers and this effect was inhibited by HC030311 as well as by TRPA1 knockout. Topical H_2_S application increased meningeal blood flow in the rat and this effect was sensitive to both NOS inhibitor, l-NMMA, and TRPA1 antagonist. In addition, systemic administration of HC030311 or CGRP_8–37_ increased blood pressure in the rats, and hypotensive effect of H_2_S was impaired in TRPA1 knockout or CGRP knockout mice [67]. Finally, H_2_S in solution at relatively low concentration (10 μM) increased CGRP release from perivascular afferent endings of the mouse mesenteric artery. H_2_S-induced relaxation of the mesenteric artery was abolished by l-NMMA, HC030311 and CGRP_8–37_, as well as by capsaicin-induced CGRP depletion. Taken together, these data strongly suggest that HNO originating by the interaction of endogenously formed NO and H_2_S increases CGRP release from perivascular sensory neurons to induce vasorelaxation [68,69]. Although this mechanism cannot operate in endothelial cells, HNO can be formed in these cells through the reaction of H_2_S and NO and then induced relaxation of adjacent smooth muscle cells [61].

## 9. Conclusions and Future Directions

Although H_2_S is produced in vascular smooth muscle cells and can directly regulate vascular tone in the autocrine manner, H_2_S synthesized in endothelial cells may have specific roles and specific targets, such as small and intermediate-conductance calcium-activated K^+^ channels or endothelial NO synthase. Endothelial H_2_S production may be regulated independently of smooth muscle cells by mediators binding to endothelial cell receptors, such as acetylcholine or leptin. Endothelial H_2_S signaling is up-regulated in some pathological conditions including obesity and ischemia-reperfusion injury, which may be a specific protective mechanism maintaining the regulation of vascular tone.

The mechanism of vascular effect of H_2_S is controversial with opposite data sometimes provided by different studies. These discrepancies result most likely from using different animal species, different H_2_S donors and their concentrations, as well as experimental conditions, such as buffer composition or oxygen levels. Due to small volume of endothelial cells and huge H_2_S production in other layers of the vascular wall studying endothelial H_2_S is a challenge. Hopefully, recent development of H_2_S-sensitive fluorescent probes will result in significant progress in this field. Another important research problem is that there are no good tools to inhibit endogenous H_2_S production since currently available inhibitors have limited membrane permeability, potency and specificity. The most important challenges for future research include the regulation of endothelial H_2_S generation and signaling, changes in H_2_S synthesis in the endothelium in pathological conditions, and possible therapeutic approaches to modulate its synthesis, metabolism or signaling.
